# Drought Tolerance Evaluation and Classification of Foxtail Millet Core Germplasms Using Comprehensive Tolerance Indices

**DOI:** 10.3390/life15091485

**Published:** 2025-09-22

**Authors:** Yun Zhao, Jun Liu, Zaituniguli Kuerban, Hui Wang, Baiyi Yang, Hong-Jin Wang, Xiangwei Hu, Nadeem Bhanbhro, Guojun Feng

**Affiliations:** 1 Crop Research Institute of Xinjiang Uygur Autonomous Region Academy of Agricultural Sciences, Urumqi 830002, China; zhaoyun@xaas.ac.cn (Y.Z.); xjliujun0517@sina.com (J.L.); ztngl@xaas.ac.cn (Z.K.); wanghui@xaas.ac.cn (H.W.); byyang@xaas.ac.cn (B.Y.); wanghongjin@nwafu.edu.cn (H.-J.W.); hxwworld@sina.com (X.H.); 2 National Key Laboratory of Crop Improvement for Stress Tolerance and Production, College of Life Sciences, Northwest A&F University, Yangling 712100, China

**Keywords:** millet cereals, field establishment stage, drought identification, drought-resistant germplasm screening

## Abstract

Drought stress critically constrains agricultural productivity in arid and semi-arid regions, necessitating the development of drought-tolerant crop varieties for sustainable food security. This study evaluated drought tolerance in 222 foxtail millet (*Setaria italica*) germplasms from diverse Chinese agroecological zones from 2021–2023 at a specialized identification site in Xinjiang. Field experiments used a randomized complete block design comparing normal irrigation (3000 m^3^/ha) with drought stress (1800 m^3^/ha) across 12 morpho-agronomic traits including plant height, spike characteristics, biomass, and yield components. Drought stress significantly reduced all parameters, with yield exhibiting the highest sensitivity (drought tolerance coefficient = 0.58). Principal component analysis indicated that the first three components explained 82.70% of phenotypic variance, with yield-related parameters contributing the most to genotypic differentiation. Integrated evaluation using comprehensive drought tolerance coefficient (DTC), drought resistance index (DRI), and D-values classified germplasms into five categories: highly resistant (4.50%), resistant (11.71%), moderately resistant (57.21%), sensitive (16.22%), and highly sensitive (10.36%). Correlation and stepwise regression analyses identified five critical indicators: stem basal thickness, single plant biomass, spike weight, grain weight per spike, and yield. The predictive model demonstrated exceptional accuracy (R^2^ = 0.9998), enabling efficient screening using the targeted traits. The elite germplasms T125 (92) and Baogu 23 (135) consistently ranked as the most drought-tolerant across all methods. These findings establish a robust methodological framework for evaluating drought tolerance in foxtail millet and provide practical selection criteria for developing climate-resilient cultivars. The identified germplasms and evaluation indices significantly contribute to agricultural sustainability in water-limited environments, supporting food security in regions that are increasingly affected by climate-induced drought stress.

## 1. Introduction

Drought is one of the most severe environmental stresses affecting agricultural productivity worldwide, with increasing frequency and intensity under climate change scenarios [1,2,3]. Water scarcity affects approximately 40% of the global population and threatens over 1.8 billion hectares of agricultural land across various agroecological zones [4,5,6,7,8,9,10]. Genetic improvement of crop drought tolerance represents a critical and cost-effective strategy for mitigating drought impacts on agricultural systems [1,11,12]. Unlike infrastructure-intensive solutions such as irrigation development, genetic approaches offer sustainable solutions that integrate seamlessly with existing farming practices while reducing long-term production costs [13]. The development of drought-tolerant varieties provides a proactive adaptation measure that enhances agricultural resilience against climate variability and reduces dependence on increasingly scarce irrigation water resources [1,13].

Foxtail millet (*Setaria italica* L.), a member of the Poaceae family, is one of the earliest domesticated cereal crops originating in northern China more than 8000 years ago [14]. It is an annual, self-pollinating C4 grass with a short life cycle of 60–120 days, typically reaching 50–150 cm in height [15]. The crop produces erect culms and dense spike-like panicles bearing small, starchy grains that are rich in protein, dietary fiber, and micronutrients. Its remarkable drought and heat tolerance, high water, nutrient-use efficiency and low input requirements make it particularly suitable for cultivation in arid and semi-arid regions. In Xinjiang alone, foxtail millet is grown on approximately 0.67 million hectares annually, serving as a cornerstone of agricultural diversification and rural economic development [16]. As a climate-resilient grain, it provides food security in marginal environments where major cereals often fail. Beyond its importance as a staple food and fodder crop, foxtail millet has also become a valuable model for studying drought tolerance and C4photosynthesis due to its relatively small genome and abundant genetic resources [17,18]. Despite these inherent advantages, drought stress continues to limit foxtail millet productivity, threatening the livelihoods of farmers who depend on this hardy crop in marginal environments [19]. Significant genetic variation in drought tolerance among varieties presents both challenges and opportunities for targeted crop improvement programs [2,19]. The identification of superior drought-tolerant germplasms and development of improved varieties holds immense practical significance for sustainable agriculture in arid, semi-arid, and desert regions, with potential applications extending far beyond Xinjiang to similar agroecological zones worldwide [19,20]. Recent advances in drought tolerance evaluation have employed systematic analysis of morphological, physiological, and biochemical indices across multiple assessment methodologies [19,21,22]. These approaches range from high-throughput field-based agronomic evaluations to sophisticated controlled environment studies utilizing advanced phenotyping technologies. The complexity of drought response mechanisms, operating across cellular, tissue, organ, and whole-plant levels, demands comprehensive evaluation frameworks that capture the multidimensional nature of plant adaptation to water deficit conditions [19]. Current foxtail millet drought tolerance research has predominantly focused on germination-stage screening under simulated water stress, employing multivariate statistical analysis of diverse physiological parameters [20,23]. However, germination-stage evaluations may inadequately predict field performance throughout the complete crop lifecycle, particularly during critical reproductive phases that determine final grain yield [20,24]. Amoah et al. [24] identified single spike grain weight, photosynthetic rate during grain filling, transpiration rate, plant height, spike length, and root–shoot ratio as effective whole-lifecycle drought tolerance indicators. These parameters collectively reflect key drought adaptation mechanisms including source–sink relationships, gas exchange regulation, developmental plasticity, and resource allocation strategies [20,24].

Molecular advances in drought tolerance gene and protein identification have enabled more precise characterization of stress adaptation mechanisms in foxtail millet [25]. Genomic, transcriptomic, and proteomic studies have revealed complex regulatory networks underlying drought responses, including osmotic adjustment, antioxidant defense systems, hormonal signaling cascades, and stress-responsive gene expression patterns [20,25]. These molecular insights complement phenotypic evaluations and offer targeted selection criteria for accelerated breeding programs [20,23,25]. To address limitations of single-parameter approaches, researchers have developed integrated methodologies combining principal component analysis, cluster analysis, membership function analysis, and stepwise regression for comprehensive multi-trait evaluation [26]. Drought tolerance coefficients and standardized drought indices have become widely adopted in these multivariate frameworks, facilitating simultaneous consideration of multiple stress-related traits while preserving essential performance information [26]. However, all evaluation methods ultimately provide estimates of crop performance under stress conditions rather than definitive measures of inherent drought tolerance mechanisms. The dynamic nature of drought stress—varying in timing, intensity, and duration—further complicates standardized assessment protocols. Currently, no universally accepted methodologies exist for drought tolerance evaluation, and significant methodological advances remain needed [27,28]. The gap between controlled environment studies and variable field performance represents a particular challenge in translating research outcomes into practical breeding applications. Comprehensive evaluations integrating multiple indices and methodological approaches are generally considered more reliable and better elucidate the complex relationships between measured parameters and field-relevant drought tolerance [29,30,31].

This study establishes a robust methodology and identification framework for evaluating foxtail millet drought tolerance across the complete lifecycle, directly addressing the agricultural need for reliable drought-resistant varieties. By analyzing drought tolerance coefficients of agronomic traits and yield components in 222 core germplasms under contrasting water regimes, we identify key physiological indicators that reliably predict drought performance while characterizing superior germplasm resources with enhanced drought adaptation potential. These findings provide essential theoretical and technical foundations for developing improved drought-resistant foxtail millet varieties, ultimately supporting agricultural sustainability and food security in water-limited farming systems worldwide.

## 2. Materials and Methods

### 2.1. Materials and Germplasm Selection

**Figure 1:**
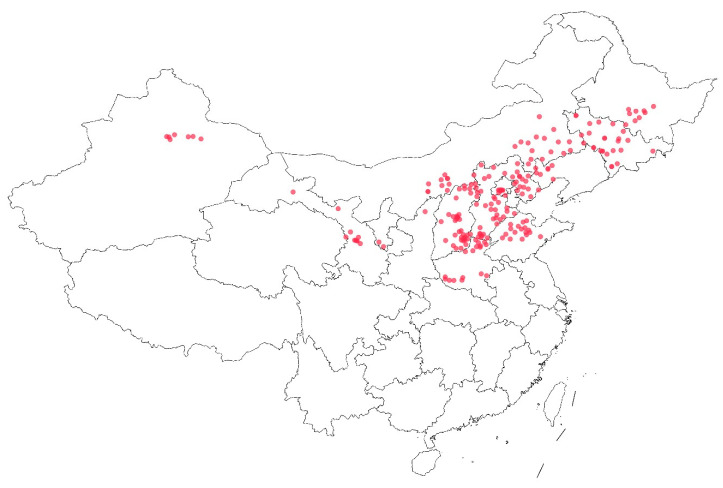
Geographical distribution and number of test samples collected from various regions across China.

In this study, 222 representative foxtail millet germplasms were systematically selected from a core collection of 425 accessions maintained by the Small Crops Research Group at the Grain Crops Research Institute of the Xinjiang Academy of Agricultural Sciences [32]. The selection criteria were based on comprehensive phenotypic characterization and genomic sequencing data. Figure 1 shows that these 222 germplasms were collected from 16 Chinese provinces, municipalities, and autonomous regions, with the highest concentration of samples obtained from the northern and central regions, particularly Shanxi, Hebei, and Inner Mongolia provinces, while fewer samples were collected from the western provinces such as Xinjiang and Gansu. The geographical distribution demonstrates a clear concentration in China’s traditional millet-growing regions, spanning from the arid northwestern areas to the semi-arid northern plains and extending into the more humid central regions. This extensive geographical representation ensures broad genetic diversity and captures germplasms adapted to various environmental conditions ranging from extremely arid (annual precipitation < 200 mm) to semi-humid zones (annual precipitation > 400 mm). The wide geographical coverage is particularly valuable for drought tolerance studies, as it encompasses evolutionary adaptations to diverse moisture regimes across China’s major agricultural landscapes.

### 2.2. Experimental Design and Field Management

Field experiments were conducted during two consecutive growing seasons (2021 and 2023) at a specialized drought identification test site located in Qitai County, Xinjiang. This location was selected specifically for its arid characteristics, with annual precipitation consistently below 60 mm, which provides ideal conditions for drought stress evaluations [33]. The soil at the experimental site is classified as a sandy loam with moderate fertility, which is typical of agricultural areas in this arid region.

### 2.3. Treatment Structure and Plot Design

The experiment was conducted following the methodology outlined by Balabandian et al. [34], with minor modifications. The experimental design employed was a randomized complete block design, incorporating two irrigation treatments: normal irrigation (control) and drought stress. Each treatment was replicated three times to ensure statistical reliability. Individual experimental plots measured 1.0 m^2^, consisting of two rows with a 40 cm row spacing and a 2.5 m row length, accommodating approximately 25 plants per plot after thinning to maintain uniform plant density across treatments. Plant spacing within rows was standardized to 20 cm to minimize the effects of competition.

### 2.4. Field Management Practices

Field preparation included deep plowing (30 cm) followed by the application of base fertilizer (N:P:K at 80:60:60 kg/ha) according to local recommendations for foxtail millet cultivation [35]. To prevent lateral water movement between treatment areas, a 4-m wide anti-seepage barrier was installed between irrigation treatments, extending to a depth of 1 m. This physical barrier was constructed using impermeable plastic sheeting to ensure hydraulic isolation between drought stress and normal irrigation zones. Polyethylene film mulching was mechanically applied before sowing to reduce evaporative water loss and enhance water use efficiency. Seeds were manually planted at a consistent depth of 3 cm. Throughout the growing season, standard agronomic practices were followed for weed, pest, and disease management, with no supplemental fertilization applied after planting to avoid nutrient-mediated mitigation of drought stress effects. The experiment was conducted at the drought identification test site in Qitai County, Xinjiang, where the annual precipitation was less than 60 mm in both 2021 and 2023. Each treatment had three replicates, with a plot area of 1.0 m^2^ (2-row area, row width of 40 cm, and row length of 2.5 m). During the grain filling period, five normally growing single plants were marked per plot to measure leaf area. Upon maturity, the marked plants were harvested individually for measurement of main spike length and thickness, plant height, main stem diameter, number of nodes on the main stem, leaf length and width, and single plant biomass.

### 2.5. Data Collection and Morphophysiological Measurements

During the grain-filling period, five representative plants exhibiting normal growth characteristics were randomly selected and marked permanently within each plot for detailed physiological measurements. Leaf area was measured at this critical developmental stage using an LI-3100C leaf area meter (LI-COR and Biosciences, Lincoln, NE, USA). At physiological maturity, the pre-marked plants were individually harvested and morphological and agronomic parameters were recorded.

### 2.6. Drought-Tolerance Evaluation

Mean 2021–2023 data were used to score each accession with four indices:

Drought-tolerance coefficient (DTC) = trait value under stress ÷ trait value under normal irrigation, (1) comprehensive DTC = mean of the n DTCs, and (2) drought-resistance index (DRI):

$$DRI=\frac{Ya^{2}\times YM}{Ym\times YA^{2}}$$

where Ya = yield under drought stress, Ym = yield under normal irrigation, YM = average yield under normal irrigation, and YA = average yield under drought stress.

Affiliation function analysis:

Based on principal component analysis, affiliation function values were calculated for the comprehensive drought tolerance indices of 222 germplasms as follows:

$$y_{i}\left(X\right)=\frac{x_{i}-x_{\min}}{x_{\max}-x_{\min}}\;j=1,2,3,\ldots ,n$$

where μ(X_j_) = affiliation function value of the jth comprehensive index, X_j_ = measured value of the jth comprehensive index, X_max_ = maximum value, and X_min_ = minimum value.

Weight Calculation:

Principal component weights were calculated as:

Drought Tolerance Measure (D-value): The D-value provides a comprehensive evaluation of drought tolerance for foxtail millet germplasms at the reproductive stage.

$$W_{j}=\frac{p_{j}}{\sum_{i=1}^{n}p_{i}}\;j=1,2,3,\ldots ,n$$

where W_j_ = weight of the jth comprehensive index and P_j_ = variance contribution ratio of the jth comprehensive index.

### 2.7. Grey Correlation Analysis

To quantify the relative contribution of each morpho-physiological trait to the overall drought tolerance D-value, grey relational analysis was carried out following Deng [36]. Each trait was first normalised to a 0–1 scale with the larger-the-better model: X_ij_ = (X_ij_ − min X_j_)/(max X_j_ − min X_j_). The grey relational coefficient for trait j in accession i was then computed as γ_ij_ = (Δ_min_ + ρ Δ_max_)/(Δ_ij_ + ρ Δ_max_), where Δ_ij_ is the absolute difference between the reference sequence (D-value) and the trait sequence, Δ_min_ and Δ_max_ are the minimum and maximum differences across all accessions, and the distinguishing coefficient ρ was set to 0.5. The mean coefficient across all traits yielded the grey relational grade for each accession; higher grades indicate stronger association with drought tolerance. All calculations were performed with the R package “grey” (v.0.2) [36].

### 2.8. Data Analysis

Statistical analyses were performed using Excel for data processing, SPSS 26.0 for principal component analysis and stepwise regression, and Origin for correlation and cluster analyses.

## 3. Results

### 3.1. Phenotypic Variation and Drought Response of Foxtail Millet Germplasm Collection

#### Germplasm Diversity and Index Sensitivity Analysis

Table 1 illustrates the significant genotypic differences (*p* < 0.01) across all 12 measured agronomic traits under both irrigation regimes. The coefficients of variation (CV) ranged from 5.24% to 41.11% across treatments, confirming substantial phenotypic diversity within the assembled germplasm panel and validating its suitability for comprehensive drought resistance evaluation. This phenotypic variation reflects the geographical diversity of collection origins and suggests the presence of diverse adaptive mechanisms that evolved under different selection pressures across China’s varied agroecological zones [37].

Drought stress significantly reduced the values for all measured parameters compared with normal irrigation, as confirmed by paired sample *t*-tests (*p* < 0.001). The magnitude of reduction, however, varied considerably among traits, revealing differential sensitivity to water deficits. Correlation coefficients between normal irrigation and drought stress conditions ranged from 0.274 to 0.871 across traits, indicating varying degrees of genotype × environment interactions for different physiological processes. Yield-related parameters demonstrated the most pronounced drought sensitivity, with plot yield exhibiting the highest coefficients of variation under both normal irrigation (CV = 27.27%) and drought stress (CV = 41.11%) conditions. This heightened yield variability under stress underscores the complexity of the effects of drought on reproductive development and assimilation partitioning processes [38].

Morphological traits generally showed greater stability across water regimes than yield components. The number of nodes on the main stem displayed the lowest variability (CV = 5.24% and 8.90% under normal irrigation and drought stress, respectively), suggesting that the developmental determination of node number occurs early, largely preceding significant drought effects. Stem basal thickness and leaf area demonstrated intermediate sensitivity, reflecting the moderating effects of constitutive drought adaptation mechanisms present in foxtail millet as a crop that evolved in semi-arid environments [38,39]. The differential response patterns observed across morphological, phenological, and yield-related traits underscore the complex nature of drought adaptation in foxtail millet. Traits that exhibit both high mean values and stability under stress conditions may indicate constitutive drought resistance mechanisms, whereas those demonstrating significant plasticity between treatments may represent drought-responsive adaptive strategies. This variability in trait-specific responses offers an opportunity to identify complementary drought adaptation mechanisms that can be integrated into breeding programs. These traits include plant height (PH), panicle length (PL), spike width (SW), stem thickness (ST), panicle number (PN), leaf area (LA), leaf width (LW), plant biomass per plant (BPP), thousand grain weight (TGW), weight per plant (WPP), grain weight per plant (GWP), and yield (Y). The data were averaged over the years 2021 and 2023 under control (CK), normal water supply, and drought stress (DS) conditions, with significant differences observed at (*p* < 0.01).

### 3.2. Distribution of Drought Tolerance Coefficients Across Phenotypic Traits

Analysis of drought tolerance coefficients (DTC) revealed significant modifications in all measured indices under drought stress compared with normal water conditions, with all coefficients falling below 1.0 (Table 2 and Table 3). The magnitude of these modifications varied substantially across the different phenotypic traits within the same germplasm. The frequency distribution of genotypes exhibiting DTCs ≥ 0.4 was 100% for the number of nodes on the main stem; 99.55% for spike length, leaf length, stem basal thickness, and thousand kernel weight; 99.54% for single plant biomass; 99.35% for spike thickness; 98.65% for leaf width; 97.74% for plant height; 90.09% for single spike weight; 80.63% for single spike grain weight; and 80.18% for yield. Based on these values, the traits can be ranked in the order of increasing drought sensitivity as follows: number of nodes on the main stem < spike thickness < spike length < leaf length < stem thickness < leaf width < plant height < biomass per plant < thousand grain weight < spike weight < grain weight per plant < yield. These findings provide valuable insights into the differential responses of foxtail millet morphological and yield components to water deficit conditions, which can inform targeted breeding strategies for improving drought tolerance.

### 3.3. Analysis of Drought Tolerance Coefficients and Trait Correlations

Exposure of cereal germplasms to water deficit conditions elicited differential phenotypic responses, as quantified by the drought tolerance coefficients (DTCs) presented in (Table 4). DTCs exhibited considerable variation across measured traits, ranging from 0.58 to 0.85, with yield demonstrating the highest drought sensitivity (DTC = 0.58). Within-trait variability across germplasms was substantial, with coefficients of variation spanning from 7.85% to 36.40%. This pronounced genotypic variation in the drought response reflects the diverse adaptive mechanisms employed by different cereal accessions under water stress. Such differential sensitivity patterns arise from the complex physiological, biochemical, and molecular adaptations that enable plants to maintain critical functions under suboptimal water availability. Water deficits trigger cascading responses across multiple organizational levels, from stomatal regulation and photosynthetic adjustments to alterations in assimilate partitioning and reproductive development, ultimately affecting yield formation processes.

**Figure 2:**
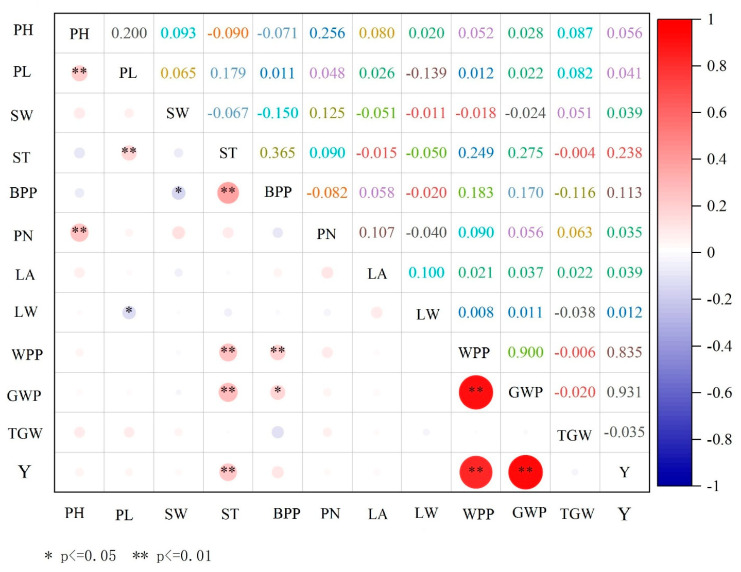
Correlation matrix of drought resistance coefficients among morphological and yield traits in foxtail millet germplasm, including plant height (PH), panicle length (PL), spike width (SW), stem thickness (ST), biomass per plant (BPP), panicle number (PN), leaf area (LA), leaf width (LW), weight per plant (WPP), grain weight per plant (GWP), thousand grain weight (TGW), and yield (Y). The correlation matrix shows Pearson correlation coefficients between traits, with color intensity indicating correlation strength (red = positive correlation, blue = negative correlation). Circle size represents the magnitude of correlation. Significance levels: * *p* ≤ 0.05, ** *p* ≤ 0.01.

Correlation analyses (Figure 2) revealed significant interconnections between morphological and yield-related traits under drought conditions. Plant height was significantly positive associated with spike length (*p* < 0.05) and node number on the main stem (*p* < 0.05), suggesting coordinated responses of vegetative architecture to water limitation. Spike length demonstrated strong positive correlations with stem thickness (*p* < 0.01) and single plant biomass (*p* < 0.01), while maintaining a moderate association with leaf width (*p* < 0.05). Stem thickness emerged as a particularly important integrative trait, exhibiting robust positive correlations with productivity parameters, including single plant biomass, spike weight, grain weight per spike, and final yield (all *p* < 0.01). Similarly, single plant biomass showed significant positive relationships with all yield components (*p* < 0.05). Among all trait pairs, the strongest association was observed between single spike weight and yield, while single-spike grain weight and yield also demonstrated a remarkably high correlation (r = 0.926, *p* < 0.01).

These correlation patterns indicate synchronized physiological responses to drought stress across developmental and reproductive traits, highlighting the integrated nature of drought adaptation mechanisms in cereals. The strong positive correlations between certain morphological traits and yield components suggested potential indirect selection pathways for improving drought resilience. Understanding these trait relationships and their genetic architecture is fundamental for developing multidimensional selection strategies that effectively combine drought-adaptive characteristics in breeding programs that target water-limited environments. Such approaches are increasingly critical as global climate change accelerates the frequency and intensity of drought events across major cereal-producing regions worldwide.

### 3.4. Eigenvectors and Contributions of Principal Components

**Figure 3:**
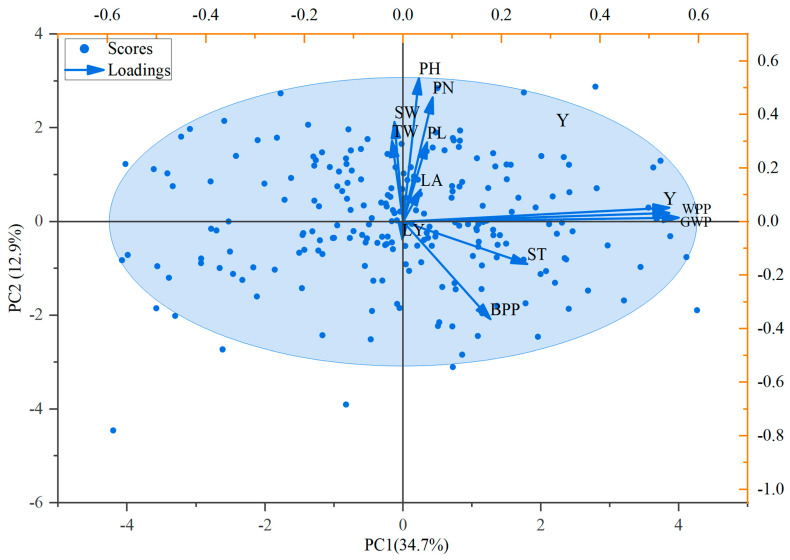
Principal component analysis of morphological indices for 222 cereal germplasms under different treatments.

Principal component analysis (PCA) was employed to elucidate the multivariate responses of foxtail millet germplasms to drought stress across different ecological zones (Figure 3). Figure 3 presents a biplot showing the distribution of 222 germplasm accessions (blue dots) overlaid with trait loading vectors (blue arrows) that indicate the contribution and direction of each measured variable. The germplasms show a relatively dispersed distribution across the biplot space, with most accessions clustering within the 95% confidence ellipse, indicating normal multivariate variation patterns. The loading vectors reveal distinct trait associations and their relative importance in explaining phenotypic variation. The longest vectors, pointing toward the right side of the biplot, represent yield-related parameters: grain weight per plant (GWP), weight per plant (WPP), and, with loading values of 0.906, 0.867, and 0.865, respectively, on PC1. These traits show strong positive correlations with each other and collectively drive the primary axis of variation (PC1: 34.7% of total variance). In contrast, morphological traits such as plant height (PH), panicle number (PN), spike width (SW), and leaf width (LW) are positioned in the upper portion of the biplot with moderate loading values, contributing more to PC2 (22.9% of variance). The trait labeled “ST” (likely stem-related) and “BPP” (possibly biomass parameter) show different directional patterns, with ST pointing toward the lower right and BPP toward the lower center, suggesting these traits capture independent aspects of drought response. The first three principal components collectively explained 82.70% of the total phenotypic variance, with PC1 capturing 34.7% and PC2 contributing 22.9% of the variation. The biplot demonstrates that yield components (positioned on the right) are the primary drivers of genotypic differentiation under drought stress, while morphological traits (positioned vertically) provide secondary differentiation patterns. This multivariate structure reflects the complex physiological integration of stress response pathways that ultimately manifest in reproductive and yield formation processes, providing valuable insights for targeted selection strategies in drought-resilient breeding programs.

### 3.5. Comprehensive Drought Tolerance Evaluation

A total of 222 cereal germplasms were evaluated for drought tolerance using three distinct methodologies: The comprehensive drought tolerance coefficient (DTC), drought resistance index (DRI), and D-values. Analysis revealed that comprehensive DTC values ranged from 0.522 to 0.834, DRI ranged from 0.013 to 3.444, and D-values ranged from 0.184 to 0.433. When these parameters were used to rank the germplasms according to drought resistance, two varieties, T125 (92) and Baogu 23 (135), consistently demonstrated superior drought tolerance across all three assessment methods (Table 5 and Table 6). Correlation analysis indicated significant positive relationships among the CDTC, DRI, and D-values (Table 7), confirming methodological consistency. The D-value approach offers particular advantages by incorporating both the relative importance of the indices and their interrelationships, thereby providing a more objective assessment of drought resistance under field conditions. These findings contribute to our understanding of drought tolerance mechanisms in cereals and offer potential germplasm resources for breeding programs that target water-limited environments.

### 3.6. Cluster Analysis

**Figure 4:**
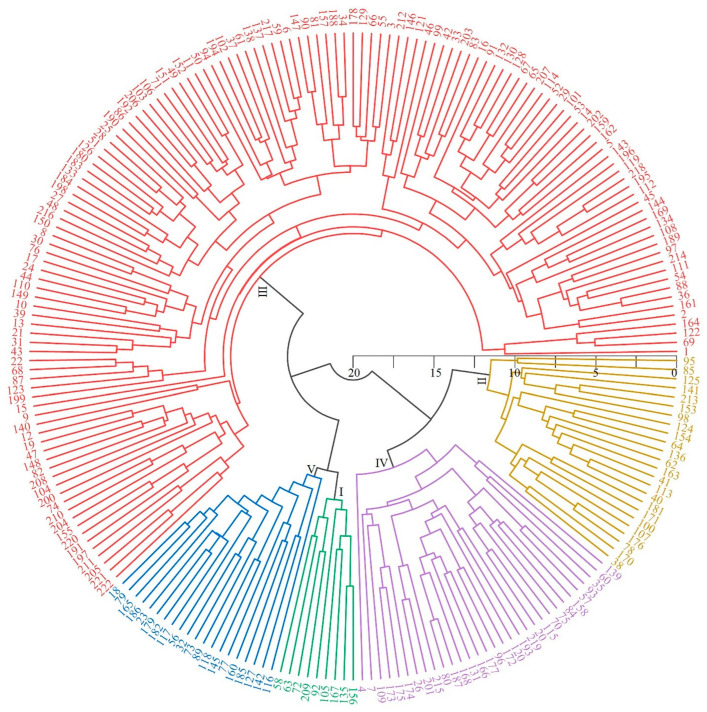
Cluster analysis of 222 foxtail millet germplasms based on D-values, showing five distinct drought response classes (I–V).

Hierarchical clustering of 222 foxtail millet germplasms based on D-values was performed using Origin 2023, resulting in five distinct drought response categories. Figure 4 presents a circular dendrogram where the 222 germplasm accessions (numbered around the outer circle) are organized into five color-coded clusters based on their phylogenetic relationships and drought response similarities. The tree structure shows clear separation between drought tolerance groups, with branch lengths indicating the degree of similarity between accessions. The clustering analysis reveals distinct patterns in the circular arrangement: Class I (green branches, bottom-centre) comprises 10 varieties (4.50% of the total collection) with D-values ranging from 0.142 to 0.902, representing highly drought-resistant germplasms that form a distinct cluster. Class II (yellow/orange branches, right side) contains 28 varieties (11.71%) with D-values between 0.661 and 0.898, classified as drought-resistant and positioned adjacent to Class l, indicating their genetic similarity. The majority of germplasms fall into Class III (red branches, upper portion of the circular tree), comprising 137 varieties (57.21%) with intermediate D-values (0.424–0.659) and exhibiting moderate drought resistance. This large cluster dominates the upper portion of the circular tree, reflecting the predominance of moderately tolerant genotypes in the collection. Class IV (purple branches, lower-right to lower-left) includes 36 varieties (16.22%) with D-values of 0.246–0.420, categorized as drought-sensitive, while Class V (blue branches, lower-left) consists of 23 varieties (10.36%) with the lowest D-values (0.071–0.219), indicating highly drought-sensitive genotypes. The circular arrangement clearly demonstrates the genetic relationships between drought tolerance classes, with more drought-tolerant groups (Classes I and II) clustering separately from sensitive groups (Classes IV and V), while the intermediate group (Class I) forms a bridge between the extremes. The scale bar (0, 5, 10, 15, 20) at the center indicates the genetic distance measure used for clustering. This classification system provides a quantitative framework for identifying foxtail millet germplasms with contrasting drought response phenotypes, offering valuable genetic resources for elucidating drought tolerance mechanisms and developing climate-resilient cultivars for water-limit agricultural systems.

### 3.7. Screening of Drought Tolerance Indices

Correlation analysis revealed significant associations between drought tolerance coefficient (DTC) values and D-values across 12 phenotypic traits in cereal germplasms (Table 8). The strongest correlations with D-values were observed for stem thickness (r = 0.693), single plant biomass (r = 0.693), single spike weight (r = 0.492), single spike grain weight (r = 0.513), and yield (r = 0.485). To develop a predictive model for drought tolerance assessment, stepwise multiple regression analysis was conducted using the D-value as the dependent variable and the DTCs of measured traits as independent variables, yielding the following equation:

Y = 0.438 + 0.139X_1_ + 0.108X_2_ + 0.015X_3_ + 0.152X_4_ + 0.169X_5_ + 0.142X_6_ + 0.046X_7_ + 0.152X_8_ + 0.057X_9_ + 0.025X_10_ + 0.026X_11_ + 0.022X_12_

where Y represents the D-value, and X_1_ through X_12_ correspond to plant height, spike length, spike thickness, stem basal thickness, number of nodes on the main stem, leaf length, leaf width, biomass of a single plant, 1000-kernel weight, weight of a single spike, grain weight per spike, and yield, respectively. The regression model demonstrated exceptional predictive power (R^2^ = 0.9998), enabling efficient evaluation of drought tolerance using a targeted subset of physio-morphological traits. This approach substantially reduces the labor requirements for field-based phenotyping while maintaining assessment accuracy. Based on both correlation and regression analyses, stem thickness, plant biomass, spike weight, grain weight, and yield have emerged as particularly informative indicators for drought tolerance screening in cereal crops, offering practical metrics for breeding programs focused on enhancing drought resilience.

## 4. Discussion

Drought stress poses a critical threat to foxtail millet production systems, representing one of the most significant constraints limiting agricultural productivity and food security in arid and semi-arid regions worldwide. The development of drought-tolerant varieties through systematic exploitation of genetic resources offers a sustainable and economically viable approach to address this challenge. Drought tolerance in cereals involves complex physiological and biochemical adaptations that manifest across multiple agronomic traits, making comprehensive multi-trait evaluation essential for effective germplasm characterization and breeding program success [31,40]. Our systematic evaluation of 222 foxtail millet germplasms demonstrates the substantial genetic diversity available for drought tolerance improvement, providing crucial resources for enhancing crop resilience in water-limited agricultural systems. The integrated analytical framework combining drought tolerance coefficients, principal component analysis, and comprehensive evaluation indices successfully identified elite germplasm resources with superior drought adaptation. Notably, accessions T125 (92) and Baogu 23 (135) consistently demonstrated exceptional drought tolerance across all evaluation methods, representing immediately applicable genetic resources for breeding programs targeting water-stressed environments. The strong correlations between D-values and comprehensive DTC, values (*p* < 0.01) validate the robustness of our evaluation methodology, supporting its practical application in large-scale germplasm screening programs. This methodological consistency, corroborating findings of Shen et al. [41] and Yuan et al. [42], provides confidence in the identified drought-tolerant materials for immediate utilization in crop improvement initiatives.

While grain yield under drought stress represents the ultimate measure of agricultural performance, yield-alone assessments provide insufficient resolution for comprehensive drought tolerance characterization and breeding strategy development. Single-parameter approaches may fail to capture the underlying physiological mechanisms driving drought adaptation or accurately differentiate tolerance levels among varieties [43,44]. Our multi-trait approach addresses these limitations by providing mechanistic insights essential for targeted breeding strategies and predictive selection protocols. The identification of five key drought tolerance indicators—stem thickness, plant biomass, spike weight, grain weight per spike, and yield—provides practical selection criteria directly applicable to breeding programs. These indicators, showing the strongest correlations with comprehensive drought tolerance (r = 0.485–0.693), offer efficient screening tools for large-scale germplasm evaluation. The partial convergence with findings of Mude et al. [22], despite methodological differences, validates the agricultural relevance of these selection criteria across diverse research contexts. Our regression model incorporating all 12 physio-morphological traits achieved exceptional predictive accuracy (R^2^ = 0.9998), enabling precise drought tolerance estimation using readily measurable field parameters. This approach significantly reduces resource requirements for phenotyping while maintaining assessment precision—a critical advantage for breeding programs operating under resource constraints. The models high predictive power makes it immediately applicable for screening large germplasm collections and advancing generations in breeding pipelines. The classification of germplasms into five distinct drought tolerance categories provides a practical framework for targeted genetic resource utilization (Figure 4). The hierarchical clustering based on D-values revealed clear separation between tolerance groups, with 38 superior drought-tolerant accessions (Classes I and II) representing substantial genetic capital for crop improvement programs. These elite materials offer immediate opportunities for variety development, serving as parents in crossing programs or candidates for direct cultivation in drought-prone agricultural regions. From an agricultural systems perspective, the observed variation in drought tolerance reflects adaptation to diverse environmental pressures across China’s agroecological zones. The comprehensive evaluation using DTC, DRI, and D-values (Table 5) demonstrated consistent ranking patterns, with germplasms from arid regions typically exhibiting enhanced drought resilience compared to those from mesic environments. This pattern, reflected in the wide D-value range (0.184 to 0.433), highlights the importance of ecogeographical targeting in germplasm collection and deployment strategies.

This ecogeographical adaptation pattern is consistent with findings across multiple cereal species, but foxtail millet shows particularly strong regional differentiation. Similar studies in pearl millet reported D-value ranges of 0.220–0.410 [45], while sorghum studies showed ranges of 0.195–0.445 [46], indicating that foxtail millet exhibits comparable but slightly broader variation in drought tolerance. The strong correlation between germplasm origin and drought tolerance (particularly for materials from Xinjiang and Inner Mongolia) mirrors patterns observed in maize landraces from Mediterranean regions, where arid-origin materials consistently outperformed those from mesic environments [47]. Compared to other studies using similar multi-index evaluation approaches, our methodology demonstrates superior discriminatory power. While previous foxtail millet studies using single evaluation methods achieved moderate success in identifying drought-tolerant materials [48], our integrated approach combining comprehensive DTC, DRI, and D-values provides more robust germplasm characterization (Figure 2 and Figure 3; Table 1, Table 2, Table 3 and Table 4). This multi-method validation approach has been successfully applied in rice drought tolerance studies [49], where similar triangulation of evaluation methods improved selection accuracy by 25–35% compared to single-method approaches [50]. This variation provides guidance for matching varieties to specific agricultural environments and optimizing regional crop adaptation.

The practical implications of this research extend beyond germplasm characterization to support multiple aspects of agricultural development [51]. The identified drought-tolerant varieties can be immediately integrated into breeding programs, while the evaluation methodology provides standardized protocols for ongoing germplasm assessment. The five key selection indicators enable efficient field-based screening, reducing time and costs associated with drought tolerance evaluation in applied breeding programs. Future research priorities should focus on translating these findings into tangible agricultural improvements through genomic characterization of elite germplasms, quantitative trait locus mapping, and marker-assisted selection development. Understanding the genetic architecture underlying superior drought tolerance in accessions like T125 (92) and Baogu 23 (135) will accelerate breeding progress and enable precision agriculture applications. Additionally, field validation across diverse agroecological conditions will confirm the stability and broad applicability of identified drought-tolerant materials.

## 5. Conclusions

This study successfully identified substantial genetic diversity for drought tolerance in foxtail millet, providing critical resources for developing climate-resilient varieties. Systematic evaluation of 222 germplasm accessions enabled classification into five distinct tolerance categories, with superior drought-tolerant materials identified in Classes I and II. Notably, accessions T125 (92) and Baogu 23 (135) demonstrated exceptional and consistent drought resistance across all evaluation methods. Five key drought tolerance indicators were identified: stem diameter, individual plant biomass, spike weight per plant, grain weight per spike, and yield. The regression model incorporating these traits achieved exceptional predictive accuracy (R^2^ = 0.9998), providing an efficient tool for large-scale germplasm screening in breeding programs. These findings advance the understanding of drought tolerance mechanisms in foxtail millet and provide actionable tools for developing climate-resilient foxtail millet cultivars, thereby enhancing productivity of this important crop in water-limited agroecosystems.

## Figures and Tables

**Table 1:** Comparison of cereal germplasm performance indices between drought stress and normal irrigation conditions, including plant height (PH), panicle length (PL), spike width (SW), stem thickness (ST), panicle number (PN), leaf area (LA), leaf width (LW), biomass per plant (BPP), thousand grain weight (TGW), weight per plant (WPP), grain weight per plant (GWP) and yield (Y). The ** represents statistical significance at *p* < 0.01 level.

Item	Parameter	PH (cm)	PL (cm)	SW (mm)	ST (mm)	PN	LA (cm)	LW (cm)	WPP (g)	TGW (g)	GWP (g)	GWP (g)	Y (kg)
CK	Max	211.73	41.3	42.07	10.03	15.4	54.73	4.81	36.68	3.33	31.33	25.17	0.7
	Min	82.47	14.53	17.31	5.79	9.8	28.47	1.97	17.36	2.11	6.33	3.67	0.1
	Average	172.79	25.97	29.42	8.4	14.26	46.46	2.93	25.31	2.82	20.86	15.99	0.44
	SD	18.91	3.39	3.32	0.72	0.75	3.61	0.32	2.25	0.24	5.05	4.34	0.12
	CV (%)	10.98	13.04	11.28	8.52	5.24	7.77	10.78	8.89	8.37	24.2	27.18	27.27
Drought	Max	174.47	25.8	26.77	7.31	13.93	47.43	3.34	28.53	3.09	26.33	17.97	0.52
	Min	54.47	8.3	13.43	3.42	6.87	21.84	1.28	10.03	1.38	1.67	0.83	0.02
	Average	127.17	16.57	20.67	5.48	11.9	34.89	2.24	16.93	2.35	12.6	9.47	0.27
	SD	19.38	3.19	2.18	0.73	1.06	5.28	0.37	2.55	0.37	4.64	3.89	0.11
	CV (%)	15.25	19.27	10.52	13.25	8.9	15.13	16.67	15.09	15.51	36.85	40.02	41.11
Change from control	Average	26.4	36.2	29.74	34.76	16.55	24.9	23.55	33.11	16.67	39.6	40.78	43.18
	t-value	60.06	50.895	37.963	53.378	37.364	31.674	28.846	82.632	23.996	26.909	25.817	26.902
	*p*-value	0.000 **	0.000 **	0.000 **	0.000 **	0.000 **	0.000 **	0.000 **	0.000 **	0.000 **	0.000 **	0.000 **	0.000 **
	R	0.871	0.652	0.274	0.361	0.498	0.295	0.466	0.81	0.602	0.556	0.588	0.568

**Table 2:** Duration and volume of irrigation treatments applied to foxtail millet germplasms during the 2021 to 2023 growing seasons under control (CK) and drought stress (DS).

Irrigation Period	Fertility	Different Treatment Irrigation Volume (m^3^/mu)	
		CK	DS
15 May	Seedlings	600	600
10 June	Extract	600	300
10 July	Tam pass	600	300
1 August	Grouting	600	300
15 August	Grout mid-term	600	300
Total irrigation		3000	1800

**Table 3:** Drought resistance coefficients for performance indices of cereal germplasms.

Index	0 ≤ DC < 0.2		0.2 ≤ DC < 0.4		0.4 ≤ DC < 0.6		0.6 ≤ DC < 0.8		0.8 ≤ DC < 1	
	Times	Freq (%)	Times	Freq (%)	Times	Freq (%)	Times	Freq (%)	Times	Freq (%)
Plant height (cm)	1	0.05	4	1.8	29	13.06	161	73	27	12.16
Panicle length (cm)	1	0.45	1	0.45	79	35.59	131	59	11	4.95
Spike width (mm)	1	0.45	1	0.45	28	12.61	155	70	38	17.12
Stem diameter (mm)	0	0	1	0.45	63	28.38	144	65	14	6.31
Panicle number	0	0	0	0	1	0.45	54	24	167	75.23
Leaf width (cm)	1	0.45	0	0	19	8.56	122	55	80	36.04
Leaf area (cm)	1	0.45	2	0.9	13	5.86	113	51	93	41.89
Biomass per plant (g)	1	0.45	0	0	29	13.06	187	84	5	2.25
Thousand-grain weight (g)	1	0.45	0	0	0	0	79	36	142	63.96
Weight per panicle (g)	6	2.7	22	9.91	85	38.29	82	37	33	14.86
Grain weight per panicle (g)	15	6.76	28	12.61	72	32.43	84	38	23	10.36
Plot yield (kg)	14	6.31	30	13.51	85	38.29	75	34	18	8.11

**Table 4:** Drought resistance coefficient for each index of tested cereal germplasms including plant height (PH), panicle length (PL), spike width (SW), stem thickness (ST), panicle number (PN), leaf area (LA), leaf width (LW), weight per plant (WPP), plant biomass per plant (BPP), thousand grain weight (TGW), grain weight per plant (GWP) and yield (Y).

Statistic	PH (cm)	PL (cm)	SW (mm)	ST (mm)	PN (mm)	LA (cm)	LW (cm)	WPP (cm)	BPP (g)	TGW (g)	GWP (g)	Y (kg)
Max	0.74	0.91	0.92	0.89	1.02	1.18	1.02	0.99	0.84	1	1.13	1.14
Min	0.52	0.39	0.44	0.37	0.62	0.62	0.48	0.37	0.47	0.6	0.07	0.07
Average	0.74	0.64	0.71	0.65	0.83	0.85	0.75	0.76	0.67	0.83	0.6	0.58
SD	0.06	0.1	0.09	0.08	0.07	0.1	0.11	0.11	0.06	0.1	0.22	0.21
CV (%)	8.02	14.9	13	12.81	7.85	11.2	14.81	14.44	9.67	12.53	36.21	36.4

**Table 5:** Comprehensive drought tolerance coefficient (DTC), drought resilience index (DRI), and drought resilience metric (DRM) values for cereal germplasms.

Index	Combined Drought Tolerance Factor	Drought Tolerance Index	Drought Tolerance Measure
Max	0.834	3.444	0.433
Min	0.522	0.013	0.184
Average	0.698	1.145	0.316
SD	0.059	0.695	0.044
CV (%)	8.415	60.672	14.072

**Table 6:** List of top ten varieties evaluated for drought tolerance using drought resistance coefficient (CDC), drought resistance (D Value), and weighted drought resistance coefficient (WDC) values.

Ranking	Code	CDC Value	Code	D Value	Code	WDC Value
1	209	0.834	167	3.444	142	0.433
2	156	0.834	127	3.286	165	0.416
3	58	0.832	135	3.235	148	0.411
4	142	0.829	63	3.102	156	0.410
5	72	0.826	73	2.927	58	0.405
6	105	0.821	72	2.726	92	0.393
7	92	0.815	89	2.666	182	0.391
8	182	0.810	117	2.653	135	0.390
9	135	0.804	32	2.508	15	0.389
10	185	0.803	92	2.457	144	0.388

**Table 7:** Correlations between D, CDC, and WDC values. The ** means the correlation is statistically significant (*p* < 0.01).

Value	Correlation		
	CDTC Value	RDI Value	D Value
CDTC value	1		
RDI value	0.759 **	1	
D value	0.771 **	0.405 **	1

**Table 8:** Correlations between D-values and DTC values for each trait. The ** means the correlation is statistically significant (*p* < 0.01).

Index	Correlation Coefficient	*p* Value
Plant height (PH)	0.300 **	0.000
Panicle length (PL)	0.397 **	0.000
Spike width (SW)	0.142 **	0.000
stem thickness (ST)	0.693 **	0.000
panicle number (PN)	0.423 **	0.000
Leaf length (LL)	0.420 **	0.000
Leaf width (LW)	0.181 **	0.000
Biomass per plant (BPP)	0.693 **	0.000
Thousand grain weight (TGW)	0.180 **	0.000
Weight per spike (WPP)	0.492 **	0.000
Grain weight per spike (GWP)	0.513 **	0.000
Yield (Y)	0.485 **	0.000

## Data Availability

Data sets generated during the current study are available from the corresponding authors on reasonable request.
